# scSemiGCN: boosting cell-type annotation from noise-resistant graph neural networks with extremely limited supervision

**DOI:** 10.1093/bioinformatics/btae091

**Published:** 2024-02-16

**Authors:** Jue Yang, Weiwen Wang, Xiwen Zhang

**Affiliations:** School of Mathematics, Sun Yat-sen University, Guangzhou 510000, China; Department of Mathematics, School of Information Science and Technology, Jinan University, Guangzhou 510000, China; Department of Bioinformatics, College of Medical Information Engineering, Guangdong Pharmaceutical University, Guangzhou 510000, China

## Abstract

**Motivation:**

Cell-type annotation is fundamental in revealing cell heterogeneity for single-cell data analysis. Although a host of works have been developed, the low signal-to-noise-ratio single-cell RNA-sequencing data that suffers from batch effects and dropout still poses obstacles in discovering grouped patterns for cell types by unsupervised learning and its alternative–semi-supervised learning that utilizes a few labeled cells as guidance for cell-type annotation.

**Results:**

We propose a robust cell-type annotation method scSemiGCN based on graph convolutional networks. Built upon a denoised network structure that characterizes reliable cell-to-cell connections, scSemiGCN generates pseudo labels for unannotated cells. Then supervised contrastive learning follows to refine the noisy single-cell data. Finally, message passing with the refined features over the denoised network structure is conducted for semi-supervised cell-type annotation. Comparison over several datasets with six methods under extremely limited supervision validates the effectiveness and efficiency of scSemiGCN for cell-type annotation.

**Availability and implementation:**

Implementation of scSemiGCN is available at https://github.com/Jane9898/scSemiGCN.

## 1 Introduction

Single-cell RNA sequencing (scRNA-seq) which measures signals of genetic molecular at cell resolution enables cell-type stratification to reveal cell heterogeneity, hence allows to uncover cell lineages and composition of complex tissues, providing detailed landscapes of cell fate trajectories and progress of diseases in organism (Tang *et al.* 2009, Treutlein *et al.* 2014, Han *et al.* 2020). Undoubtedly, exploring scRNA-seq data is fundamental to achieve such biological understanding and clinical applications (Lähnemann *et al.* 2020, Wu and Zhang 2020).

Cell-type annotation that distinguishes different types of cells is a vital step in scRNA-seq data analysis. Traditional annotation methods first build unsupervised learning models to divide cells into subgroups according to the underlying difference in scRNA-seq data, then find the marker genes for each group with differential expression analysis. By matching marker genes with known cell types, subgroups are labeled with annotations (Wu and Zhang 2020). Clustering is the key step in the pipeline, and a few of works have been proposed to process scRNA-seq data (Grün *et al.* 2015, Levine *et al.* 2015, Macosko *et al.* 2015, Wang *et al.* 2017). For example, SIMLR learned similarities between cells via multiple kernel learning, then applied spectral clustering to discover subgroups (Wang *et al.* 2017). SAFE-clustering integrated outcomes of four popular clustering methods by hypergraph partitioning algorithms to obtain a consensus result (Yang *et al.* 2019). With the advantages of deep models in representation learning, a number of cell-type annotation methods based on deep networks have been developed. Wang *et al.* (2021a) proposed a deep learning framework that iterated within multiple auto-encoders to learn graph embedding of cells, and then obtained cell clusters by *k*-means and Louvain. Tian *et al.* (2019) trained an auto-encoder with both zero-inflated negative binomial loss and KL-divergence to learn low-dimensional embeddings and clustering assignment simultaneously.

Although unsupervised methods are label-free, they require expertise to find marker genes or reference databases for a specific cell type (Shao *et al.* 2021). Decoupling clustering and annotating in the learning process may also lead to biologically meaningless subgroups. Cell-type annotation methods based on semi-supervised learning emerge as an economic way to tackle these issues (Kim *et al.* 2019, Chen *et al.* 2021, Wei and Zhang 2021, Dong *et al*. 2022, 2023, Xu *et al.* 2022, Seal *et al.* 2023). Dong *et al.* (2023) employed word2vec to learn gene embeddings which were fed into branch bidirectional LSTM networks with a shared module. Then they trained their model with labeled and unlabeled data in a multi-task learning manner. Similarly, Xu *et al.* (2022) proposed scSemiGAN which consisted of generative adversarial networks and a decoder to obtain cell-type identities and latent representations of cells using labeled data as additional supervised signals. Wei and Zhang (2021) proposed to annotate unlabeled cells by alternatively updating a logistic regression model and spectral clustering as the former acted as a predictive model while the latter generated pseudo labels of cells under the consistency constraint. These semi-supervised cell-type annotation methods show promising results, but they can be further improved under the consideration of quality of scRNA-seq data.

Owing to technical artifacts of scRNA-sequencing, scRNA-seq data are contaminated with high level of noise caused by sequencing depth, experimental designs, and operations, etc. (Lähnemann *et al.* 2020). Apart from technical issues, cell-type annotation is also plagued with biological challenges. For examples, transient biological states bring ambiguity in cell-type identification (Kiselev *et al.* 2019), and skewed distributions of cell types make it difficult to capture patterns of rare categories. Thus, using raw scRNA-seq data directly for analysis generally leads to unsatisfactory outcomes. To ensure trustworthy discoveries, efforts have been made to handle batch effects and dropout of scRNA-seq data (Huang *et al.* 2018, Wei and Li 2018, Korsunsky *et al.* 2019, Yu *et al.* 2023).

In this paper, we present a semi-supervised method based on multi-layer graph convolutional networks (GCN) (Kipf and Welling 2017) called scSemiGCN for cell-type annotation. GCN has been employed for scRNA-seq data analysis for its outstanding ability to capture complex and high-order connections in networks (Wang *et al.* 2021a,b, Gao *et al.* 2023, Lewinsohn *et al.* 2023). By representing cells as nodes in a network, holistic topological relationship between cells is built by messages passing in a forward GCN. The adjacent matrix in GCN, usually constructed by scRNA-seq data to depict the relationship between cells, is crucial in transmission of information. But it may be unreliable due to the low signal-to-noise ratio of scRNA-seq data, and thus impairs the learning process, while few have taken it into account.

To address this issue, we apply SIMLR to learn similarities between cells and subsequently employ Network Enhancement (NE) (Wang *et al.* 2018) as a denoising procedure that diminishes suspicious connections and strengthens forceful links. By replacing the two-sided normalized transmission matrix with the denoised similarity matrix that achieves favorable eigengap in GCN, scSemiGCN ensures a discriminative structure in the cell-to-cell network, which helps to improve the predictive power. Additionally, to achieve better representations of nodes in the network as initial features for GCN, the raw scRNA-seq data are projected to a discriminative representation space by supervised contrastive learning (Khosla *et al.* 2020), where cells from the same types lie close and the different are far apart. To this end, all cells should be annotated beforehand. scSemiGCN preliminarily generates pseudo labels for unlabeled cells by *k*-nearest neighbors (KNN) leveraging the denoised similarity matrix and only a few labeled cells. With the advantage of an enhanced network structure and discriminative initial features, we can finally attain a powerful two-layer GCN for cell-type annotation prediction learned with a small number of annotated cells.

**Figure 1.**
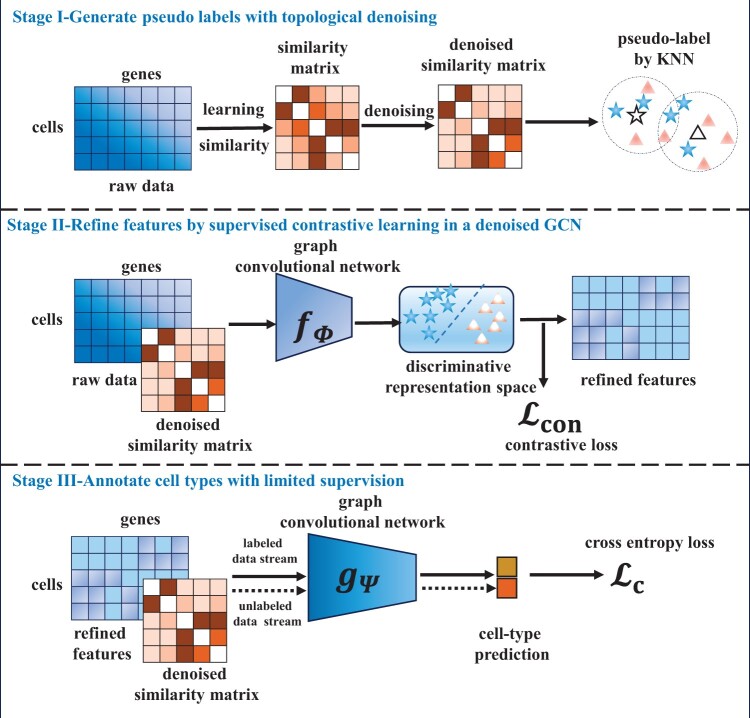
Framework of scSemiGCN. It consists of three stages: (i) generating pseudo labels for unannotated cells with denoised similarities in *k*-nearest neighbors (KNN); (ii) projecting raw features onto a discriminative representation space by supervised contrastive learning; (iii) training a cell-type annotation model with labeled cells in a two-layer graph convolutional network (GCN) using refined features and the denoised network structure as inputs.

To summarize, our contributions are as follows:

- We propose scSemiGCN consisted of topological denoising and feature refinement to handle low signal-to-noise-ratio scRNA-seq data for semi-supervised cell-type annotation. The framework of scSemiGCN is shown in Fig. 1.
- By applying a denoising procedure to cell-to-cell similarities, we obtain a more reliable network structure from which we generate pseudo labels and build a denoised GCN.
- We then refine scRNA-seq data by supervised contrastive learning with pseudo labels using the denoised GCN as backbone. Sequentially, we learn a denoised GCN for cell-type annotation with refine features and extremely limited supervision.
- We evaluate scSemiGCN in six real scRNA-seq datasets and a more challenging continuum dataset by comparing with semi-supervised and unsupervised methods. Experimental results show its competitive or even better performance over competing methods utilizing only five percent of labeled cells.

## 2 Materials and methods

### 2.1 Notations and preliminaries

We denote a scRNA-seq expression matrix as $X\in R^{m\times n}$ that contains expression signals of *n* genes of *m* cells. The lowercase bold symbol $x_{i}\in R^{n}$ denotes the expression signals of cell *i*. Suppose there are *m_l_* annotated cells and *m_u_* unannotated cells. The genomic expression matrix ***X*** can be denoted as $X=[X_{l};X_{u}]$ where $X_{l}\in R^{m_{l}\times n}$ and $X_{u}\in R^{m_{u}\times n}$ are expression matrices of annotated and unannotated cells, respectively. Considering there are *c* types of cells, let $Y_{l}\in {\left\{0,1\right\}}^{m_{l}\times c}$ represent the cell-type indication matrix of $X_{l}$ where cell types of cells are denoted by one-hot coding in rows. We aim to infer the cell-type indication matrix $Y_{u}\in {\left\{0,1\right\}}^{m_{u}\times c}$ of the unannotated *m_u_* cells. Here we propose scSemiGCN to achieve this goal.

scSemiGCN consists of three stages. First, we generate pseudo labels for unannotated cells with KNN by leveraging a denoised similarity matrix. Then we refine the low signal-to-noise-ratio scRNA-seq data by projecting it onto a discriminative representation space in a supervised contrastive learning paradigm. Finally, we train a two-layer GCN with labeled cells for cell-type annotation using a more reliable topological network structure and discriminative features as input. Details are presented in the following sections.

### 2.2 Generate pseudo labels with topological denoising

**Cell-to-cell similarities**. We employ SIMLR (Wang *et al.* 2017) to learn cell-to-cell similarities. SIMLR returns a similarity matrix $S\in R_{+}^{m\times m}$ by alternating optimization:

(1) $$\begin{array}{c} \underset{S,H,w}{\min}-\sum_{i\;=\;1}^{m}\sum_{j\;=\;1}^{m}\sum_{l\;=\;1}^{L}w_{l}K_{l}(x_{i},x_{j})S_{i,j}\;+\;\beta ||S|{|}_{F}^{2}\;+\;\\ \gamma \text{trace}(H^{T}(I_{m}-S)H)\;+\;\rho \sum_{l\;=\;1}^{L}w_{l}\;\text{log}\;w_{l}\\ s.t.H^{T}H\;=\;I_{d},\sum_{l\;=\;1}^{L}w_{l}\;=\;1,w_{l}\geq 0,\\ \sum_{j\;=\;1}^{m}S_{i,j}\;=\;1,S_{i,j}\geq 0 \end{array}$$

where $I_{m}$ and $I_{d}$ are *m *×* m* and *d *×* d* identity matrices, respectively. The symbols *d*, *β*, *ρ*, and *γ* denote non-negative hyperparameters. The parameter *d* can be set as the number of desired clusters in the dataset. Both *β* and *γ* are estimated by a data-driven approach and *ρ* is set as *m*^2^. The modified Gaussian kernels with different hyperparameters are used to define a series of kernels ${\left\{K_{l}\right\}}_{l=1}^{L}$, each of which takes the form as

(2) $$K_{l}(x_{i},x_{j})=\frac{1}{\epsilon_{i,j}^{\left(l\right)}\sqrt{2\pi }}\text{exp}\;\left(-\frac{||x_{i}\;-\;x_{j}|{|}_{2}^{2}}{2{\left(\epsilon_{i,j}^{\left(l\right)}\right)}^{2}}\right).$$

The scaled parameter $\epsilon_{i,j}^{\left(l\right)}$ is computed by

(3) $$\mu_{i}^{\left(l\right)}=\frac{\sum_{o\in N_{x_{i}}^{\left(l\right)}}\left|\right|x_{i}\;-\;x_{o}|{|}_{2}}{k^{\left(l\right)}},\;\epsilon_{i,j}^{\left(l\right)}=\frac{\sigma^{\left(l\right)}(\mu_{i}^{\left(l\right)}+\mu_{j}^{\left(l\right)})}{2},$$

where $N_{x_{i}}^{\left(l\right)}$ is the top $k^{\left(l\right)}$ nearest neighbors of $x_{i}$ in Euclidean distance. By varying $\left(k^{\left(l\right)},\sigma^{\left(l\right)}\right)$, we obtain multiple kernels ${\left\{K_{l}\right\}}_{l=1}^{L}$. Following Wang *et al.* (2017), we generate 55 kernels by setting $k^{\left(l\right)}\in \{10,12,14,\ldots ,30\}$ and $\sigma^{\left(l\right)}\in \{1.0,1.25,1.50,\ldots ,2\}$.

**Topological denoising**. Apparently, kernels calculated by Equation (2) are still suspicious due to high dimensionality and high noise level of $x_{i}$, causing ambiguous neighbors constructed in Euclidean space. Hence it may lead to an undermined similarity matrix ***S***. Wang *et al.* (2017) proposed a diffusion step for ***S*** to alleviate such tendency. Alternatively, we here apply a more powerful Network Enhancement (NE) (Wang *et al.* 2018) which provides provable guarantee by spectral analysis of transition matrix in random walks. Specifically, NE defines the transition matrix $T\in R_{+}^{m\times m}$ as

(4) $$T_{i,j}\;=\;\sum_{o\;=\;1}^{m}\frac{P_{i,o}P_{j,o}}{\sum_{v\;=\;1}^{m}P_{v,o}},\text{and}\;P_{i,j}\;=\;\frac{S_{i,j}I_{\left\{j\in N_{i}\right\}}}{\sum_{o\in N_{i}}S_{i,o}},$$

where $N_{i}$ is the *k*-nearest neighbors of cell *i* with size as *K* and $I_{\left\{\cdot \right\}}$ denotes an indicator function. The similarity matrix is updated by random walks:

(5) $$S_{t+1}=\alpha T\times S_{t}\times T+(1\;-\;\alpha )T,$$

where we initialize $S_{0}$ with ***S*** returned by SIMLR, and *α* is a regularization parameter for restart. It can be shown that Equation (5) converges to an equilibrium graph, i.e.

(6) $$\underset{t\to \infty }{\lim}S_{t}=(1\;-\;\alpha )T{\left(I_{m}\;-\;\alpha T^{2}\right)}^{-1}.$$

where $I_{m}$ is a *m *×* m* identity matrix. Thus we can obtain a denoised similarity matrix as

(7) $$\tilde{S}=(1\;-\;\alpha )T{\left(I_{m}\;-\;\alpha T^{2}\right)}^{-1}.$$

Given the eigen-decomposition of the transition matrix $T=U\Sigma U^{-1}$, where Σ is a diagonal matrix with eigenvalues of ***T*** as diagonal elements and ***U*** is consisted of corresponding eigenvectors as columns, by Equation (7), we have

(8) $$\begin{array}{c} \tilde{S}=(1\;-\;\alpha )U\Sigma U^{-1}{\left(I_{m}\;-\;\alpha U\Sigma U^{-1}U\Sigma U^{-1}\right)}^{-1}.\\ =(1\;-\;\alpha )U\Sigma U^{-1}{\left(UU^{-1}\;-\;\alpha U\Sigma \Sigma U^{-1}\right)}^{-1}\\ =U((1\;-\;\alpha )\Sigma {\left(I_{m}\;-\;\alpha \Sigma^{2}\right)}^{-1})U^{-1}=U\tilde{\Sigma }U^{-1} \end{array}$$

where $\tilde{\Sigma }=(1\;-\;\alpha )\Sigma {\left(I_{m}\;-\;\alpha \Sigma^{2}\right)}^{-1}$ is a diagonal matrix with ${\tilde{\Sigma }}_{i,i}=(1\;-\;\alpha )\Sigma_{i,i}{\left(1\;-\;\alpha \Sigma_{i,i}^{2}\right)}^{-1}$. Hence, the denoised similarity matrix $\tilde{S}$ can be computed by Equation (8) instead of its iteration form Equation (5), with computational complexity as $O(m^{3})$.

From Equation (8), it is proved that $\tilde{S}$ obtains a larger eigengap than ***S*** has [cf. Lemma 3 in Wang *et al.* (2018)], thus results in a more discriminative similarity metric (or network structure).

**Preliminary annotation.** The entry of $\tilde{S}$, denoted as ${\tilde{s}}_{i,j}$, indicates the similarity between cell *i* and cell *j*. Once having a denoised similarity matrix $\tilde{S}$, we can generate pseudo labels for unannotated cells by KNN with labeled cells utilizing $\tilde{S}$ as a similarity metric. Formally, for an unlabeled cell *i*, let $NL_{k}(i)=\{\text{top}\;\;k\;\text{of labeled cells most similar to cell}\;i\}$, then the one-hot coding of pseudo label of cell *i* is

$${\tilde{y}}_{i}=\text{mode}(\{y_{j}|\text{cell}\;j\in NL_{k}(i)\}),$$

where $y_{j}$ represents the ground-truth label of cell *j* in one-hot coding and mode(·) denotes the majority voting operator. In our experiments, we simply set the size of nearest neighbors *k *=* *1.

### 2.3 Refine scRNA-seq data by supervised contrastive learning with a denoised GCN

In this section, we propose to refine scRNA-seq data ***X*** with supervised contrastive learning (SCL) using precise labels of $X_{l}$ and pseudo labels of $X_{u}$ for supervision. Each cell is projected onto a discriminative representation space where cells from the same types lie together and the different are far apart, with dimensionality unchanged. We define the projection as a one-layer GCN, i.e.

(9) $$\tilde{X}=f_{\Phi }(X)=\text{ReLU}(AX\Phi ),$$

where $\Phi \in R^{n\times n}$ is a learnable weight matrix and ReLU(·)=max(0,·).

**Vanilla GCN.** In the vanilla graph convolutional network, the two-sided normalization symmetric matrix A that reveals the topological structure of the network is defined as $A={\tilde{D}}^{-\frac{1}{2}}\tilde{A}{\tilde{D}}^{-\frac{1}{2}}$, where $\tilde{A}$ is the adjacent matrix with self-connections and $\tilde{D}$ is a diagonal matrix with its diagonal elements as ${\tilde{D}}_{i,i}=\sum_{j=1}^{m}{\tilde{A}}_{i,j}$.

**Denoised GCN.** From Equations (4) and (5), Wang *et al.* (2018) showed that $S_{t+1}$ remains as a two-sided normalization symmetric matrix in each iteration, i.e. $S_{t+1}1=1$ and $S_{t+1}^{T}=S_{t+1}$ with non-negative elements in $S_{t+1}$. Thus, the limit of $S_{t+1}$, i.e. $\tilde{S}$, is still a two-sided normalization symmetric matrix. Here, the bold symbol 1 indicates an all-one vector with dimensionality of *m*, i.e. $1={\left(1,1,\ldots ,1\right)}^{T}$. Further, the following theorem indicates the range of eigenvalues of the limit.

Theorem 1 *The eigenvalues of the equilibrium graph**Equation* (7)*fall into the range*[0,1].

*Proof.* Wang *et al.* (2018) have shown that ***T*** is positive semi-definite and two-sided normalized with non-negative entities. Let *λ* be an eigenvalue of ***T***, by the Gershgorin circle theorem, we have $\left|\lambda -T_{i,i}|\leq \sum_{j\neq i}|T_{i,j}\right|$, which implies $\lambda \leq T_{i,i}+\sum_{j\neq i}|T_{i,j}|=\sum_{j=1}^{m}T_{i,j}=1$ since T1=1 and $T_{i,j}\geq 0$. Because ***T*** is positive semi-definite, we conclude that the eigenvalues of the transition matrix T∈[0,1]. By Equation (8), the eigenvalue of $\tilde{S}$ can be represented as $\frac{(1-\alpha )\lambda }{1-\alpha \lambda^{2}}$ where *λ* denotes the eigenvalue of ***T***. Since α∈[0,1], we conclude our statement. □

In above proof, we simplify the claim in Wang *et al.* (2018) by using the Gershgorin circle theorem.

In a word, $\tilde{S}$ is a two-sided normalized symmetric matrix with eigenvalues in the range [0,1]. Thus, it can be used as A in Equation (9), since it naturally avoids numerical instability or extreme gradients in GCN (Kipf and Welling 2017) and captures the denoised network structure simultaneously. In the end, Equation (9) can be rewritten as

(10) $$\tilde{X}=f_{\Phi }(X)=\text{ReLU}(\tilde{S}X\Phi ).$$

The refined representation of cell *i* is indicated by ${\tilde{x}}_{i}$, corresponding to the *i*-th row of $\tilde{X}$.

**Supervised contrastive loss**. Once we obtain the refined representations of cells by Equation (10), the unknown parameter Φ is optimized by minimizing the supervised contrastive loss

(11) $$L_{\text{con}}=\sum_{i\in B}\frac{-1}{\left|P_{i}\right|}\sum_{j\in P_{i}}\;\text{log}\;\frac{\;\text{exp}\;\left({\tilde{z}}_{i}^{T}{\tilde{z}}_{j}/\tau \right)}{\;\text{exp}\;\left({\tilde{z}}_{i}^{T}{\tilde{z}}_{j}/\tau \right)+\sum_{k\in M_{i}}\;\text{exp}\;\left({\tilde{z}}_{i}^{T}{\tilde{z}}_{k}/\tau \right)},$$

where B denotes a set of samples in mini-batch, and $P_{i}$ and $M_{i}$ are a set of samples in the mini-batch with the same label and different labels of cell *i*, respectively, and $\tilde{z_{i}}$ is the unit normalization of ${\tilde{x}}_{i}$, i.e. $\tilde{z_{i}}={\tilde{x}}_{i}/||{\tilde{x}}_{i}|{|}_{2}$. The symbol *τ* in Equation (11) denotes the free-tuning temperature, which we set as 0.5 in our experiments, and $\left|P_{i}\right|$ counts the number of cells in $P_{i}$.

It should be noted that we only train a few steps with the supervised contrastive loss to learn the refined representations of cells in a bid to improve robustness since the labels of unannotated cells are not accurate in the current stage.

### 2.4 Annotate cell types with limited supervision

With refined representations $\tilde{X}$ and a denoised network structure $\tilde{S}$ available, we finally build a two-layer GCN for cell-type annotation, i.e.

(12) $$\hat{Y}=g_{\Psi }(\tilde{X})=\text{softmax}(\text{ReLU}(\tilde{S}\cdot \text{ReLU}(\tilde{S}\tilde{X}W_{1})\cdot W_{2}))$$

where $W_{1}\in R^{n\times h}$ and $W_{2}\in R^{h\times c}$ are learnable parameters denoted by Ψ. Here we use a denoised GCN with the same motivation as Equation (10). Each row of $\hat{Y}$ represents predicted cell-type probability distribution of cells. The model is merely optimized by the cross-entropy loss over labeled cells ${\tilde{X}}_{l}$, i.e.

(13) $$L_{c}=-\sum_{i\in B_{l}}y_{i}{\left(\text{log}\;{\hat{y}}_{i}\right)}^{T},$$

where $B_{l}$ is a mini-batch of cells with precise labels, and $y_{i}$ and ${\hat{y}}_{i}$ are ground-truth annotation of cell *i* in one-hot coding and corresponding predicted cell-type probability distribution, respectively.

## 3 Results

### 3.1 Competing methods and datasets

Four recently developed cell-type annotation methods were used for comparison with scSemiGCN, including CALLR (Wei and Zhang 2021), scSemiGAN (Xu *et al.* 2022), scSemiAE (Dong *et al.* 2022), and SIMLR (Wang *et al.* 2017). We also compared our denoised GCN with the vanilla GCN (Kipf and Welling 2017) and Graph Attention Networks (GAT) (Veličković *et al.* 2018) which have been widely used for semi-supervised node classification in graph learning. All of the competing methods are semi-supervised, except the unsupervised SIMLR. Here we used the similarity matrix return by SIMLR to annotate the unlabeled cells with KNN for comparison.

We ran our experiments with six single-cell RNA-seq datasets. Five of them, namely Buettner, Kolodziejczyk, Pollen, Usoskin, and Zeisel were taken from Wang *et al.* (2017), and the rest Cortex was created by Dong *et al.* (2022). Detail descriptions of these datasets are presented in Table 1.

**Table 1.** Detailed descriptions of scRNA-seq datasets.

Dataset	No. of cells	No. of genes	No. of populations
Buettner	182	8989	3
Kolodziejczyk	704	10 685	3
Pollen	249	14 805	11
Usoskin	622	17 772	4
Zeisel	3005	4412	9
Cortex	3005	19 972	7

### 3.2 Experimental settings

In our experiments, only five percent of cells in each dataset were supposed to have been annotated during training, and the rest were evenly divided for validation and test. The number of annotated cells used in training for each cell type in each dataset is summarized in Supplementary Table S1. Accuracy, F1-score, and the area under the ROC curve (AUC) of predicted annotations of test sets are reported.

We apply SIMLR to learn to cell-to-cell similarities with default settings in its implementation (Wang *et al.* 2017). How the choice of SMILR’s parameters affects scSemiGCN is investigated and presented in Supplementary Figs S1 and S2. The regularized parameter *α* and neighborhood size *K* in network enhancement, i.e. Equation (7), should to be tuned in experiments. We set *α* in the range of [0.4,0.5,0.6]. The range of neighborhood size *K* was set according to the average number of cells in each cell type. In Butter and Pollen, it was chosen between 18 and 20, while between 20 and 22 for the rest. The combination of *α* and *K* was determined by the highest accuracy in validation data for each dataset.

We set the number of genes as the dimension of input *n* for GCNs in all datasets except Cortex where we selected the top 2000 most variable genes as input for GCNs. The dimensionality of the hidden layer *h* in Equation (12) was fixed as 100. The size of mini-batch $B_{l}$ in Equation (13) was set as 100.

In stage II, we trained a denoised GCN in 10 epochs by supervised contrastive learning using SGD as the optimizer with learning rate as 0.05. In the final stage, we trained the two-layer GCN in 400 epochs using Adam as the optimizer, and the learning rate was set to be 0.001 in Buettner, Pollen, and Cortex, and 0.0005 for the rest.

### 3.3 Performance in cell-type annotation

We report the comparison between scSemiGCN and the competing methods for cell-type annotation under three metrics. Results are summarized in Table 2. AUC is not reported for CALLR and scSemiAE since they returned predicted labels without probability estimation. scSemiGCN demonstrates competitive and even dominant performance in all six datasets, showing its favorable robustness and adaptability. In SIMLR, we annotated cells by KNN using the learned similarities where the size of neighborhood for annotation *k* was selected in the range of {1, 3, 5} and determined by validation data. CALLR requires that there are at least two annotated samples per cell type in training. Hence, we used 10% of annotated cells when running CALLR in Pollen.

**Table 2.** Summary of evaluation metrics for each method in test data of each dataset.^a^

[ACC, F1, AUC]	Buettner	Kolodziejczyk	Pollen	Usoskin	Zeisel	Cortex
SIMLR	[0.978, 0.978, 0.990]	[0.999, 0.999, 1.000]	[0.905, 0.847, 0.923]	[0.925, 0.887, 0.933]	[0.929, 0.806, 0.940]	[0.915, 0.895, 0.970]
CALLR	[0.314, 0.289,—]	[0.961, 0.960,—]	[0.784, 0.770,—]^b^	[0.946, 0.940,—]	[0.938, 0.934,—]	[0.943, 0.942,—]
scSemiGAN	[0.512, 0.501, 0.690]	[0.994, 0.994, 0.997]	[0.932, 0.935, 0.993]	[0.959, 0.958, 0.986]	[0.896, 0.873, 0.970]	[0.950, 0.949, 0.989]
scSemiAE	[0.605, 0.512,—]	[0.976, 0.976,—]	[0.822, 0.811,—]	[0.729, 0.714,—]	[0.912, 0.900,—]	[0.940, 0.940,—]
GCN	[0.849, 0.847, 0.900]	[0.997, 0.997, 1.000]	[0.915, 0.909, 0.984]	[0.929, 0.929, 0.971]	[0.901, 0.896, 0.980]	[0.608, 0.583, 0.840]
GAT	[0.791, 0.784, 0.840]	[0.976, 0.976, 0.988]	[0.856, 0.833, 0.968]	[0.844, 0.841, 0.900]	[0.908, 0.903, 0.970]	[0.940, 0.940, 0.986]
scSemiGCN	[0.977, 0.977, 0.983]	[1.000, 1.000, 1.000]	[0.983, 0.980, 1.000]	[0.949, 0.948, 0.977]	[0.928, 0.925, 0.970]	[0.953, 0.953, 0.984]

a The best are indicated in blue font.

b We used 10% of annotated cells in training instead of 5% such that there are at least two labeled samples for each cell type.

There is obvious gap between our scSemiGCN and the rest methods in Pollen. It should be noted that there were at most two annotated cells used in training for each cell type in this dataset (see Supplementary Table S1). The result implies scSemiGCN is highly effective in this extremely limited supervision scenario.

Comparing between SIMLR and scSemiGCN, we see that scSemiGCN generally improves SMILR except in Buettner. Such improvement is particularly significant in Pollen, bought from both feature refinement and topological denoising as clarified in the following ablation studies. GCN outperforms GAT in four out of six datasets, which implies that GCN is a better graph-neural-network-based backbone than GAT for scSemiGCN.

**Figure 2.**
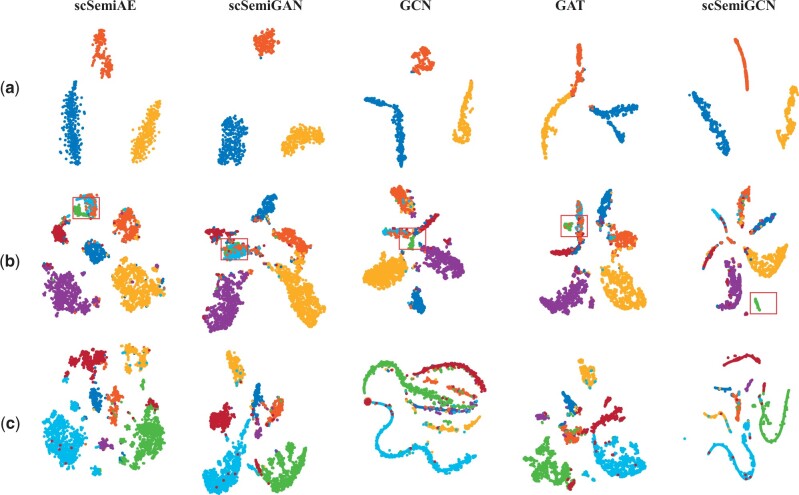
Visualization of latent representations generated by neural-network-based methods. Cell types are indicated by colors. Even there is not significant difference in separation among these methods, scSemiGCN is better at spotting a rare cell type in Zeisel indicated by red boxes. (a) Kolodziejczyk; (b) Zeisel; (c) Cortex.

We visualize latent representations generated by neural-network-based methods in three datasets with t-SNE (van der Maaten and Hinton 2008) in Fig. 2. Different categories are well separated in Kolodziejczyk of all methods. For the larger and more diverse Zeisel and Cortex, cell types with a larger proportion are more easily identified and the rare tends to be mixed with others. But we still see that the rare cell type of Zeisel indicated by green is better separated in scSemiGCN. Visualization of the rest datasets can be found in Supplementary Fig. S3.

### 3.4 Ablation studies and analysis

**Figure 3.**
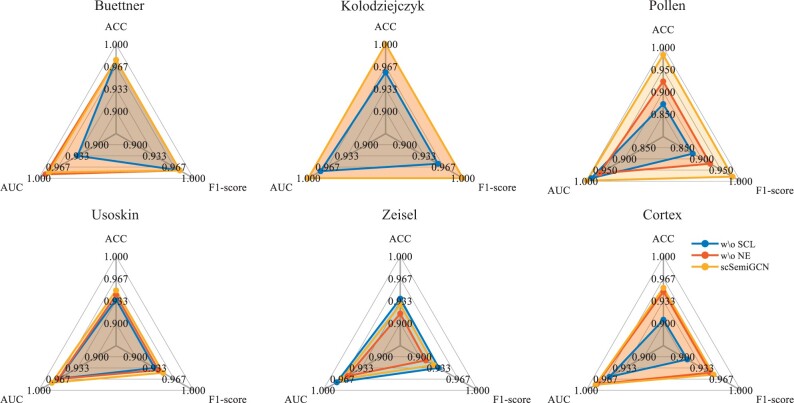
Effectiveness of feature refinement and topological denoising. We ran scSemiGCN without supervised contrastive learning (w\o SCL) and without network enhancement (w\o NE), respectively, in comparison with our full model scSemiGCN. Performance in the test of all datasets is presented. Note that it cannot tell the difference between scSemiGCN and w\o NE in Kolodziejczyk.

We also studied the effectiveness of feature refinement and topological denoising. To this end, we ran scSemiGCN bypassing stage II (withdrawing supervised contrastive learning), and denoising in stage I (withdrawing network enhancement), respectively, while keeping the remaining experimental settings unchanged. When skipping denoising in stage I, we correspondingly used vanilla GCN as the backbone instead. We present the results in Fig. 3. We can see that feature refinement helps in all datasets except Zeisel where there is marginal difference between our full model and the model without supervised contrastive learning. Network enhancement apparently boosts scSemiGCN in four out of six datasets and such improvement is significant in Pollen and Zeisel. For example, network enhancement brings nearly 10% of accuracy improvement in Pollen. We conclude that the combination of feature refinement and topological denoising delivers robustness and adaptability into scSemiGCN. Hence, we believe scSemiGCN is widely applicable to scRNA-seq data.

### 3.5 Effect of the number of annotated cells

**Figure 4.**
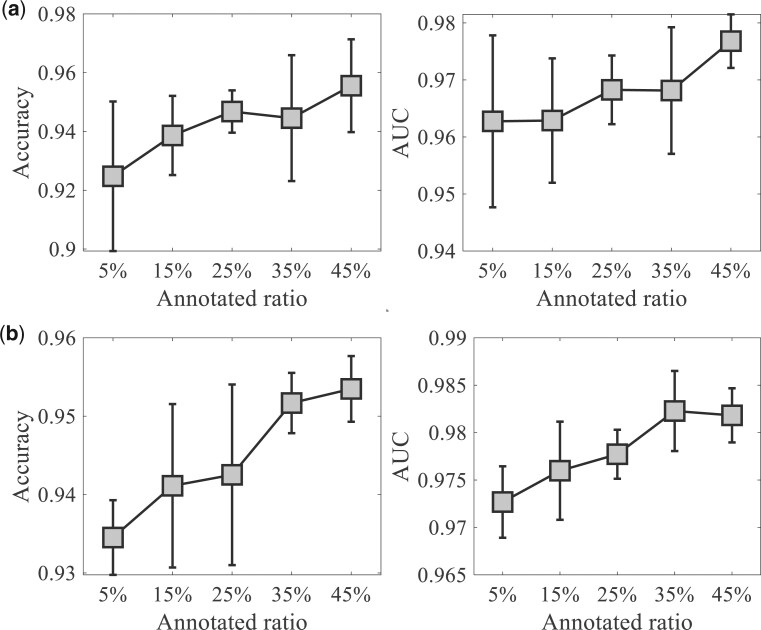
Influence of annotated ratio on the performance of scSemiGCN. For each ratio, we repeated random data split five times. Accuracy and AUC of test data are presented (mean±SD). (a) Usoskin; (b) Zeisel.

To investigate the impact of annotated proportion of cells used in training on scSemiGCN, we varied the ratio of annotated cells, ranging from 5% to 45% in training. For each annotated ratio, we repeated random data split five times. The accuracy and AUC of test data are reported. Results of Usoskin and Zeisel are shown in Fig. 4. scSemiGCN can perform better with more labeled data used for training, but it is also marginal since scSemiGCN can make good annotation using only a small proportion of labeled cells.

### 3.6 Parameter analysis

**Figure 5.**
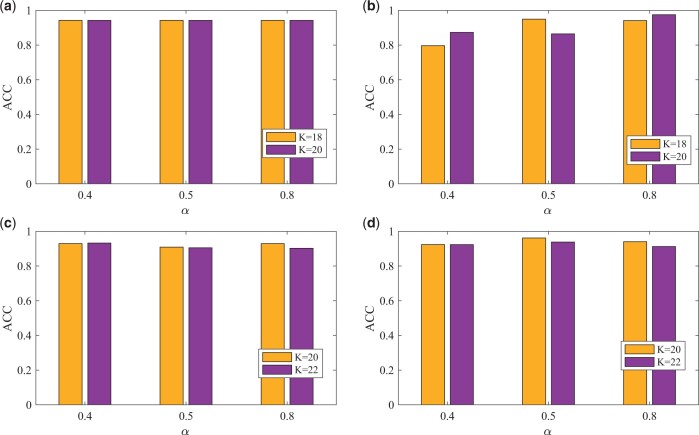
Accuracy of validation data under different settings of hyperparameters. (a) Buettner; (b) Pollen; (c) Usoskin; (d) Cortex.

Network enhancement is at the core of scSemiGCN. To study how scSemiGCN is affected by the regularized parameter *α* and the neighborhood size *K* in NE, we report accuracy of validation data under different settings of these two parameters in Fig. 5. AUC and F1-score are demonstrated in Supplementary Figs S4 and S5. We observe that scSemiGCN is more sensitive to the neighborhood size *K* in Pollen than it is in the remaining three datasets, and we posit that it is attributed to the diversity of Pollen. We also notice scSeimiGCN seems to be more stable in a larger dataset under various settings of the regularized parameter *α*, implying more efforts are needed to be taken to find an appropriate *α* for a smaller dataset.

### 3.7 Cell-type annotation for continuum immune cells

Additionally, we validated our method on a more challenging dataset built from the downsampled Tumor Immune Cell Altas (TICA) (Nieto *et al.* 2021). The constructed dataset consisted of 4223 cells from three cancer types, namely intrahepatic cholangio-(ICC), ovarian cancers (OC) and non-small-cell lung cancers (NSCLC), including 25 immune cell types. We labeled this dataset as TICA-3C. Top 2000 most variable genes were used. We followed the previously described data splitting. Only 211 labeled cells were used for training (see Supplementary Table S2) and the rest was used for validation and test. We trained scSemiGCN on TICA-3C with the same hyperparameter setup as on Cortex except the neighborhood size in NE and the learning rate for training the two-layer GCN which were set as 50 and 0.002, respectively.

**Figure 6.**
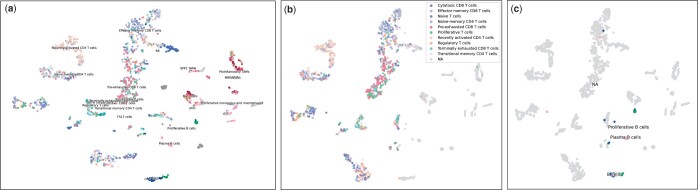
Visualization of latent representations of test data in TCIA-3C learned by scSemiGCN. Cell types are indicated by colors. (a) All 25 immune cell types; (b) T cells; (c) B cells. The rest cell types are indicated by NA in (b) and (c).

SIMLR and all deep learning methods performed poorly on the demanding TICA-3C with scSemiGCN achieving the highest accuracy 0.4432 (see Supplementary Table S3). We visualize the latent representations learned by scSemiGCN. As shown in Fig. 6, the complex and diverse T cells are mixed while the simpler B cells are better separated. Such observation is also verified by the confusion matrix (see Supplementary Fig. S6). Among T cells, the recently activated CD4 and effector memory CD8 are more easily recognized with recall as 0.669 and 0.716, respectively.

It shows that existing cell-type annotation methods may suffer in fine-grained cell-type classification and more efforts are needed to deal with such problem. Improvement from scSemiGCN shows that our method points to a potential direction.

## 4 Discussion and conclusion

In this paper, we propose a robust and well adaptive semi-supervised cell-type annotation method called scSemiGCN, based on graph convolutional networks (GCN). To achieve reliable cell-type prediction from low signal-to-noise-ratio scRNA-seq data using limited annotation cells, we employ a denoising procedure to build a trustworthy connection structure between cells, from which we obtain preliminary annotations for unidentified cells. Additionally, we refine the sequencing data by supervised contrastive learning built upon pseudo labels. Finally, we conduct message passing with refined features over the denoised topological structure of cell-to-cell network in a two-layer GCN to identify cells. Experimental results verify the effectiveness and efficiency of our method which attribute to feature refinement and topological denoising.

However, there are still a few improvements should be considered. From experimental results on TICA-3C, we see that scSemiGCN is unsatisfactory in distinguishing subtle difference between cell types lie on a continuum. Similar to all of transductive semi-supervised learning methods, scSemiGCN should be trained with all samples including labeled and unlabeled cells, which limits its application in large-scale scRNA-seq datasets. Since advances in single-cell sequencing technologies make multi-omics data available at the cell level, extending scSemiGCN to a universal model applicable to different platforms, technologies, and species will be an intriguing direction.

## Supplementary Material

btae091_Supplementary_Data

## Data Availability

Buettner, Kolodziejczyk, Pollen, Usoskin, and Zeisel were taken from Wang *et al.* (2017) available at https://github.com/BatzoglouLabSU/SIMLR. Cortex was created by Dong *et al.* (2022) available at https://github.com/PlusoneD/scSemiAE. TICA-3C was built from from the downsampled Tumor Immune Cell Altas (Nieto *et al.* 2021) available at https://zenodo.org/record/5186413#.YRqbJC1h2v6.
