# Supplementary material for: scSemiGCN: boosting cell-type annotation from noise-resistant graph neural networks with extremely limited supervision
Source: Bioinformatics. 2024 Feb 16;40(2):btae091. doi: 10.1093/bioinformatics/btae091 (PMC10904148; doi:10.1093/bioinformatics/btae091)
Supplement: btae091_Supplementary_Data [file btae091_supplementary_data.pdf]

Supplementary materials of "scSemiGCN: boosting cell-type annotation from noise-resistant graph neural networks with extremely limited supervision"

Jue Yang[1], Weiwen Wang[2]*, Xiwen Zhang[3]
[1]School of Mathematics, Sun Yat-sen University, Guangzhou, China
[2]Department of Mathematics, School of Information Science and Technology, Jinan University, Guangzhou, China
[3]College of Medical Information Engineering, Guangdong Pharmaceutical University, Guangzhou, China
* To whom correspondence should be addressed.
Email: wangww29@jnu.edu.cn.

Table S1: **The number of annotated cells for each cell type in six datasets for training.**

	Buettner	Kolodziejczyk	Pollen	Usoskin	Zeisel	Cortex
Type-1	3	15	1	7	15	11
Type-2	3	8	1	8	19	12
Type-3	3	12	1	4	47	14
Type-4	-	-	1	12	41	5
Type-5	-	-	1	-	5	41
Type-6	-	-	1	-	9	47
Type-7	-	-	1	-	10	20
Type-8	-	-	2	-	2	-
Type-9	-	-	2	-	3	-
Type-10	-	-	1	-	-	-
Type-11	-	-	1	-	-	-

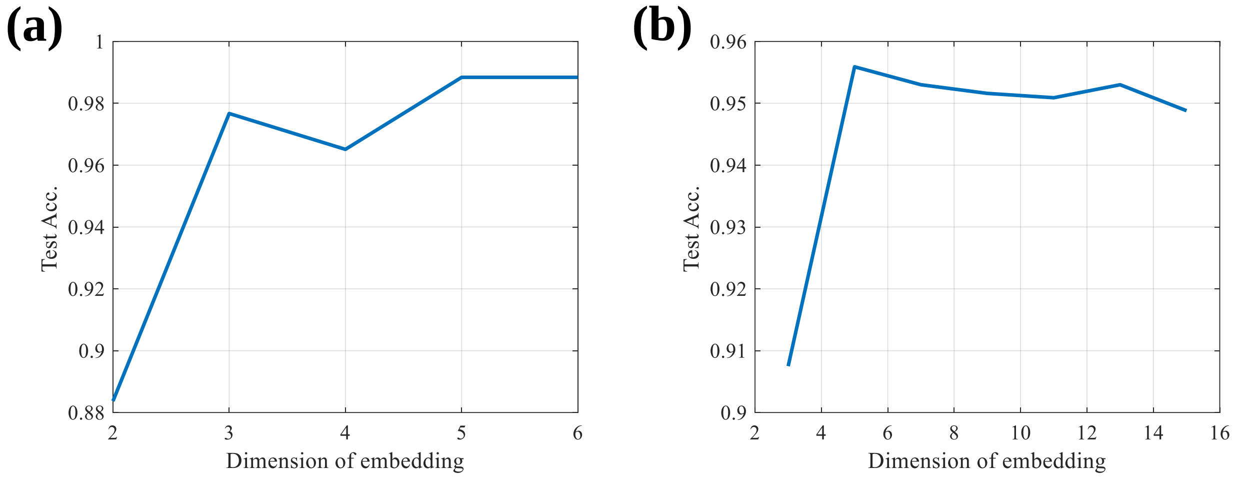

Fig. S1: **Effects of dimension of embeddings** d **in SIMLR on scSemiGCN**. (a) Buettner; (b) Cortex.

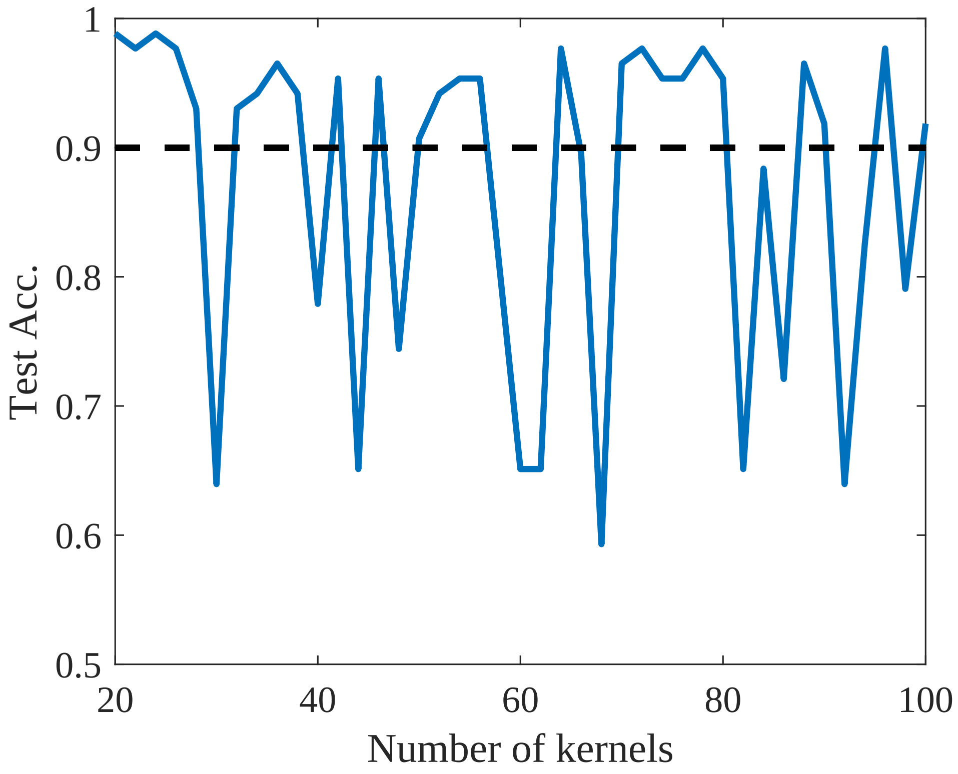

Fig. S2: **Effects of number of kernels used in SIMLR on scSemiGCN**. We take Buettner as an example. As the number of kernels varies, the accuracy oscillates. But we also see that an appropriate number of kernels can be chosen in a wide range.

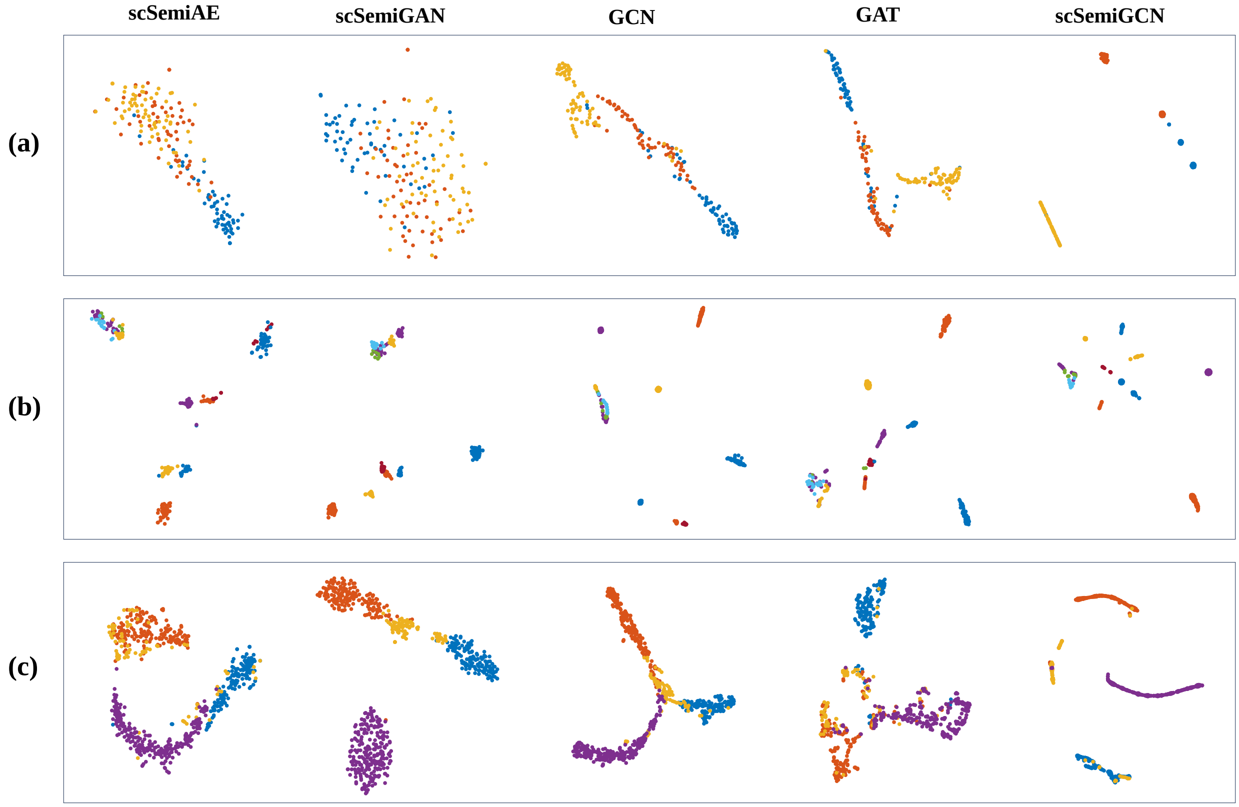

Fig. S3: **Visualization of latent representations generated by neural-network-based methods**. Cell types are indicated by colors. (a)Buettner;(b)Pollen; (c)Usoskin.

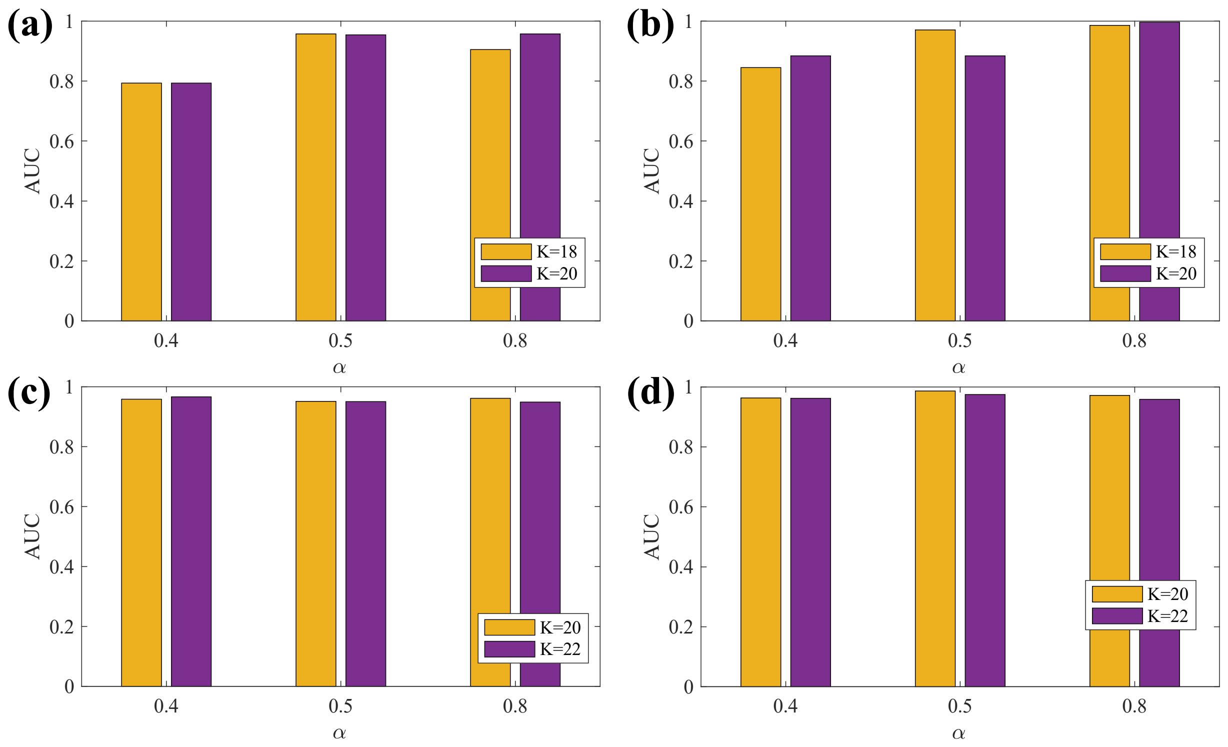

Fig. S4: **AUC of validation data under different settings of hyperparameters**. (a)Buettner; (b)Pollen; (c)Usoskin; (d)Cortex.

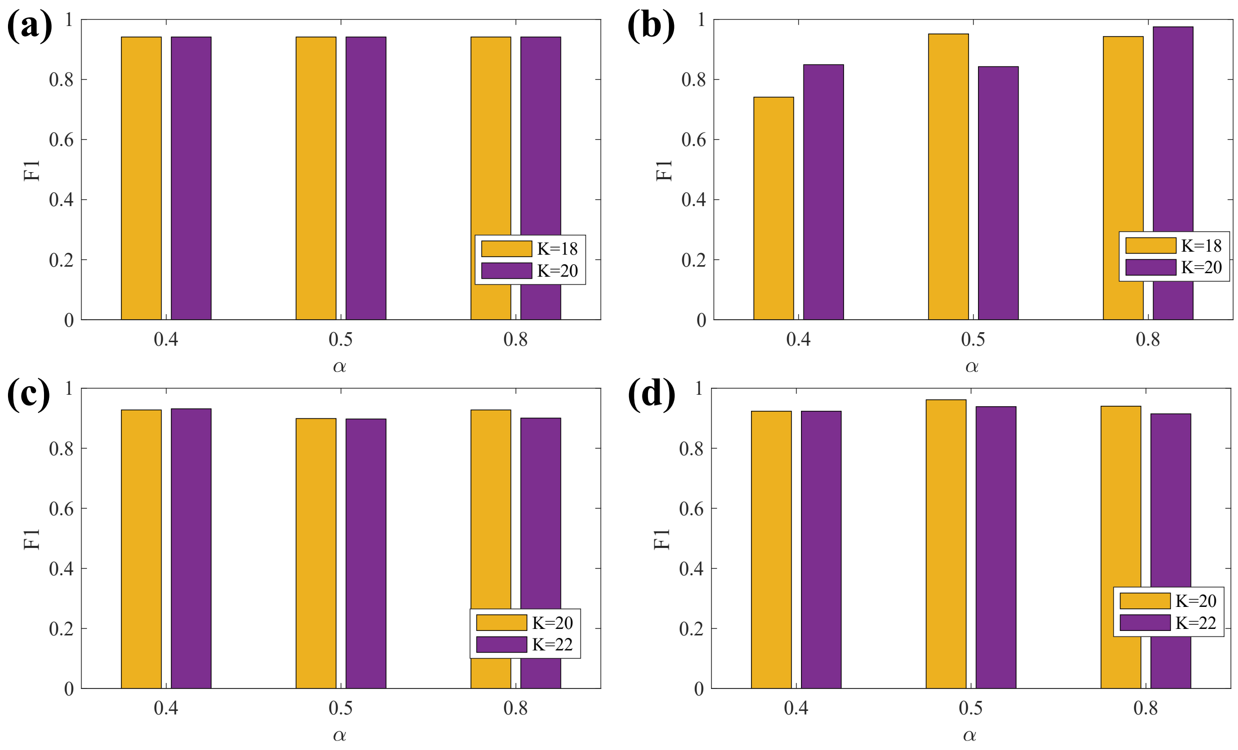

Fig. S5: **F1-score of validation data under different settings of hyperparameters**. (a)Buettner; (b)Pollen; (c)Usoskin; (d)Cortex.

Table S2: **The number of annotated cells for each cell type in TICA-3C for training.**

Cell type	Number of cells
Transitional memory CD4 T cells	7
Mast cells	4
M2 TAMs	6
T helper cells	8
Naive-memory CD4 T cells	14
Pre-exhausted CD8 T cells	17
pDC	7
Recently activated CD4 T cells	17
SPP1 TAMs	3
mDC	6
Cytotoxic CD8 T cells	9
Monocytes	5
Proinflamatory TAMs	4
Plasma B cells	4
Naive T cells	10
Effector memory CD8 T cells	16
Proliferative B cells	4
Proliferative T cells	12
NK	9
Terminally exhausted CD8 T cells	9
B cells	6
Proliferative monocytes and macrophages	4
Th17 cells	12
Regulatory T cells	12
cDC	6

Table S3: **Accuracy of test data in TICA-3C.**

	SIMLR	GCN	GAT	scSemiAE	scSemiGAN	scSemiGCN
ACC	0.3739	0.3751	0.3429	0.3721	0.3764	0.4432

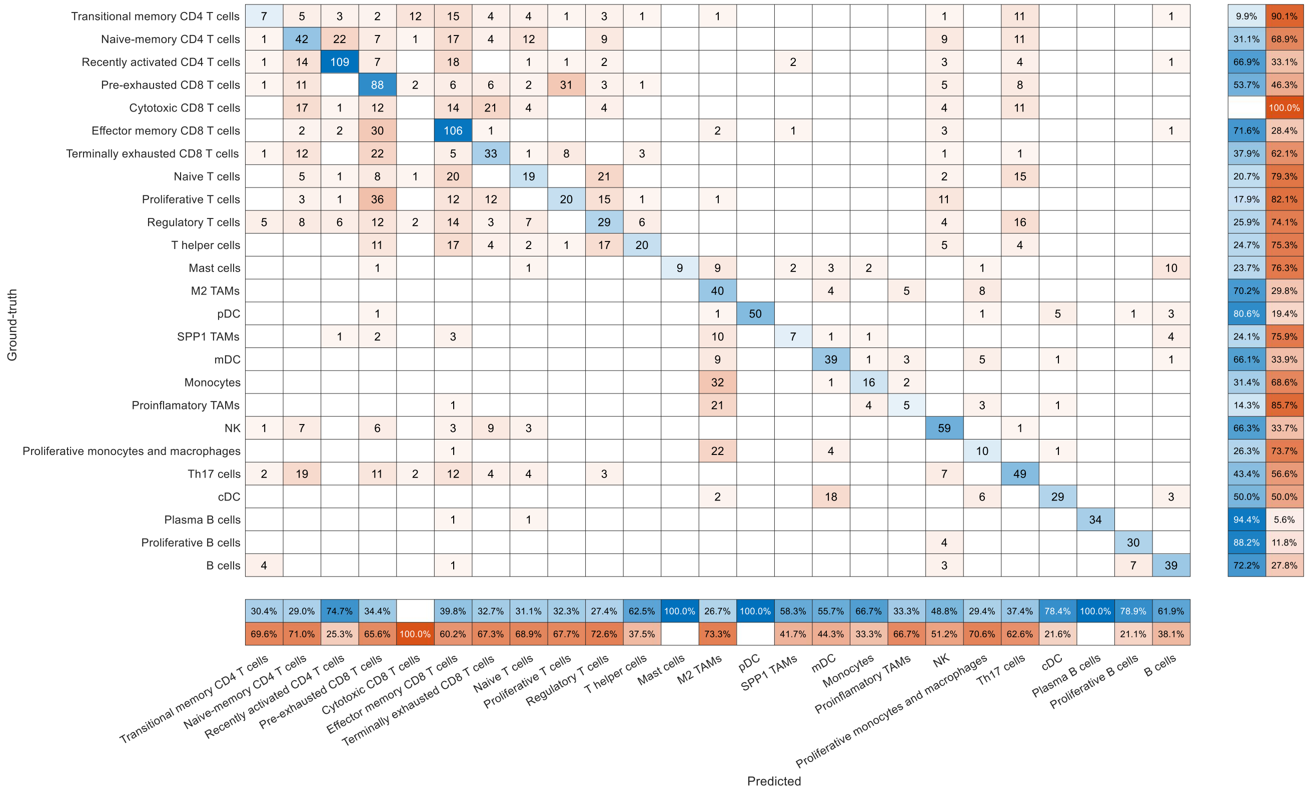

Fig. S6: **Confusion matrix of test data in TICA-3C**. Recall and precision of each cell type are also summarized in columns and rows, respectively.

Supplementary Notes

Practical usage of scSemiGCN

Semi-supervised cell-type annotation methods including scSemiGCN require a small subset of annotated cells. There are several ways to obtain such gold-standard annotated cells for semi-supervised cell-type annotation. A few as options are listed below.

- First group cells by classic clustering methods and then identify cell types of representatives of each group based on expression of marker genes.
- Project all unannotated cells onto well-labeled reference data (e.g by Seurat (Stuart *et al.*, 2021)) and select unannotated data cells with high confidence in prediction as landmarks.
- Combine representatives from well-labeled reference data (e.g Human Cell Atlas[1]) with unannotated cells.
- Utilize accumulative annotated cells.

References

Stuart, T. et al. (2019) Comprehensive integration of single-cell data. *Cell*, **177**, 1888–1902.

[1]https://www.humancellatlas.org
